# Clinical impact of visually assessed right ventricular dysfunction in patients with septic shock

**DOI:** 10.1038/s41598-021-98397-8

**Published:** 2021-09-22

**Authors:** Hiroaki Hiraiwa, Daisuke Kasugai, Masayuki Ozaki, Yukari Goto, Naruhiro Jingushi, Michiko Higashi, Kazuki Nishida, Toru Kondo, Kenji Furusawa, Ryota Morimoto, Takahiro Okumura, Naoyuki Matsuda, Shigeyuki Matsui, Toyoaki Murohara

**Affiliations:** 1 Department of Cardiology, Nagoya University Graduate School of Medicine, Nagoya, Japan; 2 Department of Emergency and Critical Care Medicine, Nagoya University Graduate School of Medicine, Nagoya, Japan; 3 Department of Biostatistics, Nagoya University Graduate School of Medicine, Nagoya, Japan

**Keywords:** Biomarkers, Infection, Infectious diseases, Cardiovascular diseases

## Abstract

We retrospectively analyzed data from the Medical Information Mart for Intensive Care-III critical care database to determine whether visually-assessed right ventricular (RV) dysfunction was associated with clinical outcomes in septic shock patients. Associations between visually-assessed RV dysfunction by echocardiography and in-hospital mortality, lethal arrhythmia, and hemodynamic indicators to determine the prognostic value of RV dysfunction in patients with septic shock were analyzed. Propensity score analysis showed RV dysfunction was associated with increased risk of in-hospital death in patients with septic shock (adjusted odds ratio [OR] 2.15; 95% confidence interval [CI] 1.99–2.32; *P* < 0.001). In multivariate logistic regression analysis, RV dysfunction was associated with in-hospital death (OR 2.19; 95% CI 1.91–2.53; *P* < 0.001), lethal arrhythmia (OR 2.19; 95% CI 1.34–3.57; *P* < 0.001), and tendency for increased blood lactate levels (OR 1.31; 95% CI 1.14–1.50; *P* < 0.001) independent of left ventricular (LV) dysfunction. RV dysfunction was associated with lower cardiac output, pulmonary artery pressure index, and RV stroke work index. In patients with septic shock, visually-assessed RV dysfunction was associated with in-hospital mortality, lethal arrhythmia, and circulatory insufficiency independent of LV dysfunction. Visual assessment of RV dysfunction using echocardiography might help to identify the short-term prognosis of patients with septic shock by reflecting hemodynamic status.

## Introduction

Sepsis is defined as life-threatening organ dysfunction due to a dysregulated host immune response to an infection, and it is associated with a high mortality rate of 30%, even in developed or developing countries^1,2^. Cardiac dysfunction in sepsis, known as septic cardiomyopathy (SCM), is common and associated with increased mortality of patients with sepsis or septic shock^3–6^. SCM has been defined as left ventricular (LV) dysfunction, such as a decrease in the LV ejection fraction^7–10^. In recent years, the effect of right ventricular (RV) dysfunction on the prognosis of patients with sepsis or septic shock has received attention^11–14^.

The effect of RV dysfunction on prognosis in sepsis or septic shock depends on population characteristics^11–14^. Management of cardiac dysfunction is considered to be important, especially in unstable patients with septic shock, but the clinical effect of RV dysfunction in this population remains unclear.

Although many parameters of RV function have been studied as prognostic indices, no consensus has been reached regarding what echocardiographic parameters should be used to define RV dysfunction. Visual estimation of RV function, which shows low interobserver variability and is a useful screening tool as a point-of-care evaluation for RV dysfunction, has not yet been evaluated in this context^15,16^. Furthermore, how RV dysfunction interacts with LV dysfunction in terms of contractility and ventricular size in patients with septic shock remains unknown.

Therefore, this study aimed to investigate the clinical significance and prognostic impact of visually assessed RV function in patients with septic shock.

## Methods

### Data source and study population

This was a retrospective observational study, and the article was prepared in accordance with the RECORD reporting guidelines^17^. We used the Medical Information Mart for Intensive Care III (MIMIC-III) version 1.4 critical care database. This is a large, freely available database comprising de-identified health-related data associated with more than 40,000 patients who stayed in the intensive care unit (ICU) of the Beth Israel Deaconess Medical Center from 2001 to 2012. The MIMIC-III database was developed and is maintained by the Laboratory for Computational Physiology at the Massachusetts Institute of Technology^18,19^.

The eligibility criteria were septic shock on ICU admission and performance of transthoracic echocardiography (TTE) within the first 24 hours of ICU admission. In accordance with the Sepsis-3 criteria^1^, septic shock was defined as hypotension with the presence of any suspected infections, the need for vasopressors, and the lactate level exceeding 2 mmol/L. Doses of other catecholamines were converted to norepinephrine-equivalent doses^20^. The decision to perform TTE was based on the clinical judgment of the medical team. Only the data of each patient’s first ICU admission were used in this study. Patients were excluded if they had the following miscellaneous etiologies of cardiac dysfunction: acute myocardial infarction, prior myocardial infarction, heart failure, infective endocarditis, aortic dissection, myocarditis, or admission to the cardiac surgery recovery unit.

### Variables and outcome measurement

In addition to the patients’ demographic data, we collected the following TTE parameters that were extracted from the echocardiography report summary data recorded by a sonographer or a cardiologist within the first 24 hours of ICU admission: visual assessment of the grade of biventricular systolic dysfunction and the degree of biventricular dilation. The quality of the TTE report, blood pressure, and heart rate at the time of TTE were also recorded. LV dysfunction was defined as an LV ejection fraction of ≤ 50% or documentation of any description of LV systolic dysfunction. The presence and grade of visual RV dysfunction were documented and categorized into two levels (i.e., mild and moderate to severe dysfunction) in line with the description in the summary reports. Other echocardiographic parameters, such as the fractional area change and tricuspid annulus systolic plane excursion, were not used as criteria of RV dysfunction because they were collected in only a few patients^14^.

Detailed hemodynamic parameters and data on fluid administration within 24 hours of ICU admission were also extracted to describe the association between visual RV dysfunction and the trend or maximum values of the hemodynamic status; the same variables acquired from different modules were combined for simplicity.

The primary outcome was in-hospital mortality. For a surrogate endpoint, the incidence of lethal arrhythmia during hospitalization was recorded. Lethal arrhythmia was defined as ventricular tachycardia or ventricular fibrillation. In addition, as a surrogate marker of systemic circulation, the trend of increasing blood lactate levels within the first 24 hours of entering the ICU was examined. An increased lactate level was defined as an increasing trend in occurrence, with a maximum after a minimum value.

### Statistical analysis

Continuous variables are expressed as median and interquartile range. Categorical variables are expressed as number (%). The relationships between LV function and RV function and between LV and RV size in patients with septic shock were visualized using chord diagrams. The hemodynamic parameters or fluid trends in association with RV function were described using locally weighted scatter plot smoothing. Non-parametric variables were compared using the Mann–Whitney U-test.

For the main analysis, propensity score analyses using inverse probability weighting methods were performed to evaluate the marginal odds of RV dysfunction for in-hospital mortality with or without the presence of LV dysfunction by adjusting for baseline imbalance^21^. To consider the severity of visually assessed RV dysfunction, additional analyses focusing on the effect of moderate to severe RV dysfunction were performed. Using a directed acyclic graph, the following confounders were used to calculate the propensity score: age, body weight, ethnicity, infection focus, SOFA score, catecholamine dosage, Elixhauser comorbidity index, mechanical ventilation, infusion fluid volume, and presence of LV dysfunction (Supplementary Fig. S1)^22^.

For sensitivity analysis, multivariate logistic regression analysis was performed to evaluate the prognostic impact of RV dysfunction and LV dysfunction. The catecholamine dosage and SOFA score were used for the covariates^23^. We also examined the interaction between RV dysfunction and respiratory failure (respiratory component of Sequential Organ Failure Assessment (SOFA) score ≥ 3 points).

Before carrying out multivariate analysis and propensity scoring analysis, multiple imputation with chained equations were performed to handle missing data, and non-normal data were log-transformed in this step. The results of 10 imputed datasets were combined with the MICE package^24^. A *P* value of < 0.05 was considered statistically significant. All statistical analyses were performed using freely available statistical software (R version 4.0.2).

### Ethics approval and consent to participate

Use of the MIMIC-III database for this study was approved by the institutional review boards of the Beth Israel Deaconess Medical Center (Boston, MA, USA) and the Massachusetts Institute of Technology (Cambridge, MA, USA) (Institutional Review Board protocol 2001-P-001699/3)^18,19^. In addition, the ethical approval statement by the local Institutional Review Board (Nagoya University Hospital Institutional Review Board) and the requirement for informed consent were waived for the use of this de-identified public database.

## Results

### Patients’ characteristics

**Figure 1:**
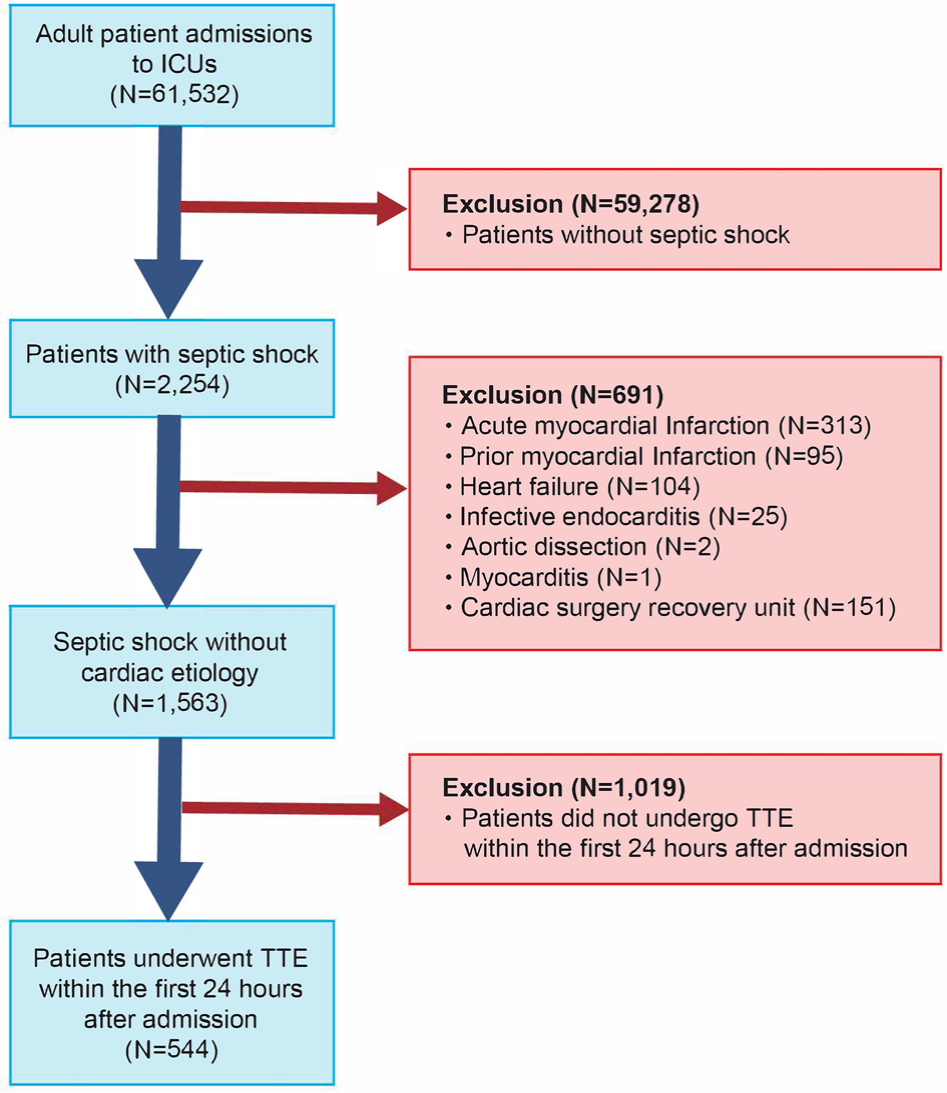
Flow diagram showing initial selection of the cohort and excluded patients. *ICU* intensive care unit, *TTE* transthoracic echocardiography.

This study included 1563 patients with septic shock. Of these patients, 544 underwent TTE (Fig. 1). The patients’ characteristics and outcomes are shown in Table 1, and the number of patients with missing data are shown in Supplementary Table S1. The patients’ median age was 67 years and the study population showed a slight male predominance (58%). The respiratory system was the most common focus of infection (39%). The median SOFA score was 13 and 40% of patients required mechanical ventilation. The median peak dosage of norepinephrine was 0.36 mcg/kg/min. LV dysfunction was present in 190 patients and RV dysfunction was present in 154. Biventricular dysfunction was present in 100 patients. A total of 235 (43.2%) patients died in the hospital. Ventricular arrhythmia developed in 41 (7.6%) patients.

**Table 1:** Patient characteristics.

Characteristics	Total (N = 544)
Age, years	67 (53–78)
Body weight, kg	81 (69–99)
Male	315 (57.9)
**Ethnicity**	
African American	43 (7.9)
Asian	13 (2.4)
Caucasian	401 (73.7)
Hispanic	16 (2.9)
Other	71 (13.1)
**Infection site**	
Abdominal	100 (18.4)
Respiratory	213 (39.2)
Soft tissue	25 (4.6)
Urinary tract	67 (12.3)
Other	33 (6.1)
Unknown	106 (19.5)
Elixhauser comorbidity index	15 (8–21)
SOFA score	13 (11–16)
Norepinephrine, mcg/kg/min	0.36 (0.14–1.05)
Lactate, mg/dL	4.0 (2.8–6.6)
Fluid administration, mL/day	4687 (1905–8050)
Mechanical ventilation	217 (39.9)
PEEP, cmH_2_O	10 (5–12)
P/F ratio	87 (60–154)
Renal replacement therapy	108 (19.9)
**Outcomes**	
Ventricular fibrillation	9 (1.7)
Ventricular tachycardia	32 (5.9)
ICU length of stay, days	6.7 (2.8–13.3)
In-hospital mortality	235 (43.2)

Data are presented as median (interquartile range) or n (%).

*ICU* intensive care unit, *PEEP* positive end-expiratory pressure, *SOFA* Sequential Organ Failure Assessment.

### Biventricular function and size

**Figure 2:**
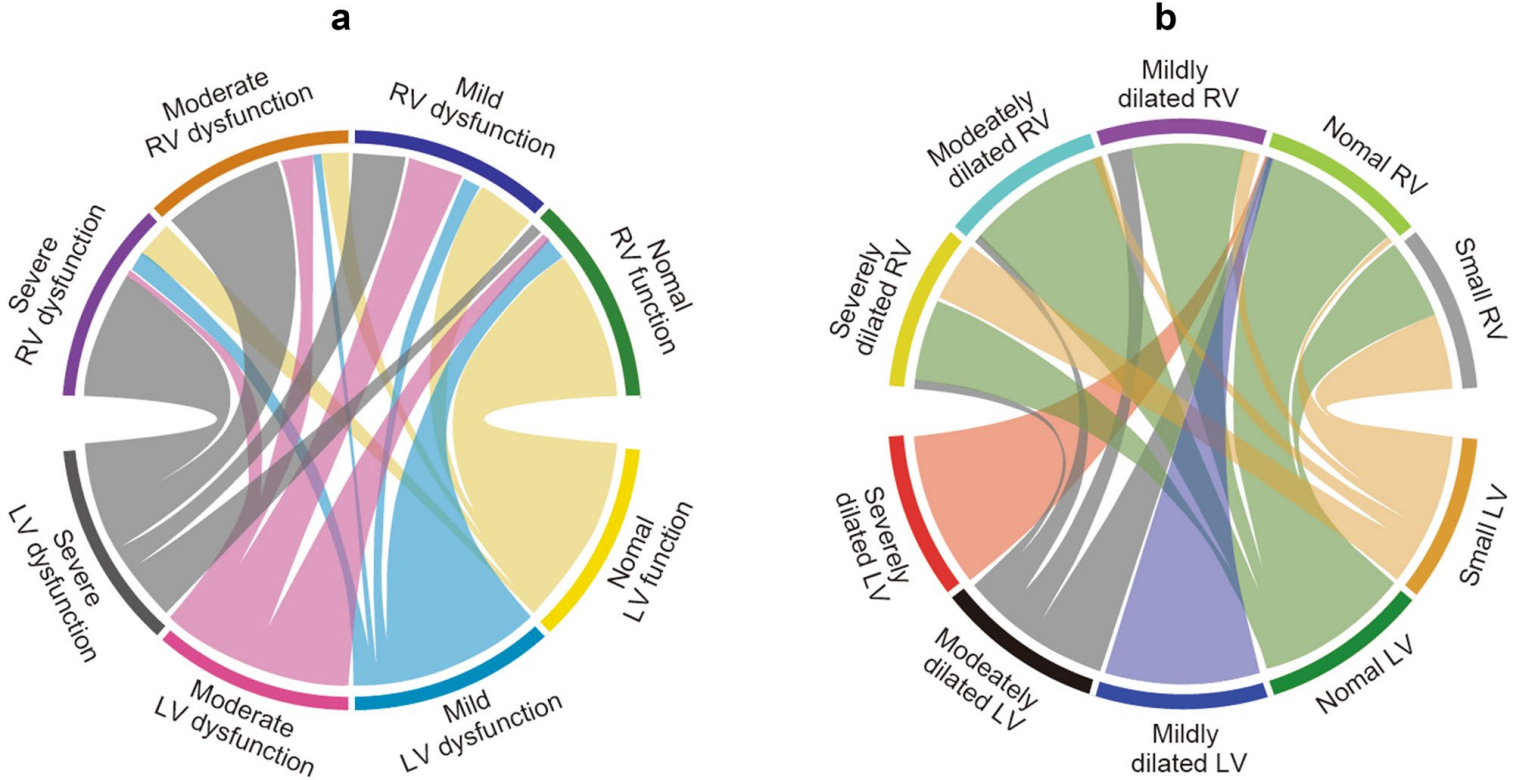
Chord diagrams. Relationships **(a)** between LV systolic dysfunction and RV dysfunction and **(b)** between LV size and RV size in patients with septic shock. *LV* left ventricle, *RV* right ventricle.

Among all patients in this study, 342 (62%) had TTE images of adequate technical quality. The details of the echocardiographic data are shown in Supplementary Table S2. The relationships between the grade of LV function and RV function and between LV size and RV size are shown in Fig. 2. The severity of RV dysfunction tended to increase as the severity of LV dysfunction increased (Fig. 2a). Conversely, the degree of LV dilation did not appear to be correlated with the grade of RV dilation, but severe RV dilation appeared to be related to a smaller LV size (Fig. 2b).

### Right ventricular function and hemodynamics

**Figure 3:**
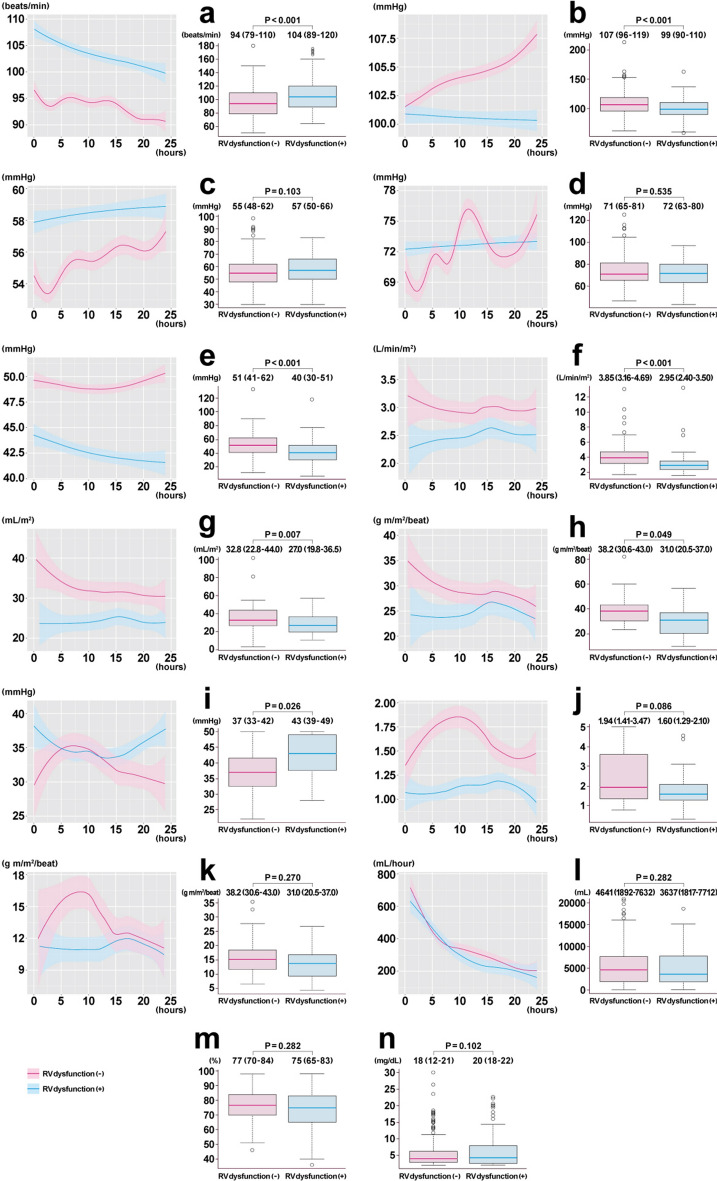
Comparison of hemodynamic parameters between patients with and those without RV dysfunction. **(a)** heart rate (n = 463), **(b)** systolic blood pressure (n = 463), **(c)** diastolic blood pressure (n = 463), **(d)** mean blood pressure (n = 463), **(e)** pulse pressure (n = 463), **(f)** CI (n = 132), **(g)** SVI (n = 97), **(h)** LVSWI (n = 48), **(i)** PAP (n = 63), **(j)** PAPi (n = 63), **(k)** RVSWI (n = 46), **(l)** infusion volume (n = 463), **(m)** ScvO_2_ (n = 364), and **(n)** lactate levels (n = 463). Data within 24 hours of ICU admission were collected. Vital signs at the time of TTE were used for comparison. The maximum values were selected in other parameters. Blood pressure was measured via an arterial catheter or noninvasively. The CI and SVI were measured by an arterial catheter, pulmonary artery catheter, pulse index continuous cardiac output monitor, or noninvasive cardiac output monitoring system. The LVSWI and RVSWI were measured by a pulmonary artery catheter, and ScvO_2_ was measured by a central venous catheter or pulmonary artery catheter. The PAPi was calculated using the following formula: (systolic PAP − diastolic PAP)/central venous pressure. *CI* cardiac index, *SVI* stroke volume index, *LVSWI* left ventricular stroke work index, *PAP* mean pulmonary artery pressure, *PAPi* pulmonary artery pulsatility index, *RVSWI* right ventricular stroke work index, *ScvO*_*2*_ central venous oxygen saturation.

Associations between visual RV function and hemodynamics are shown in Fig. 3. Patients with RV dysfunction had a higher rate of tachycardia, and had a lower pulse pressure, cardiac index, stroke volume index, and LV stroke work index compared with patients without RV dysfunction. However, the mean blood pressure and fluid administration were not different between the groups. Notably, patients without RV dysfunction had a higher RV stroke work index and pulmonary artery pulsatility index within 24 hours of their ICU stay, whereas patients with RV dysfunction did not.

### Propensity scoring analysis

The results of the propensity scoring analysis using the inverse probability of weighting method are shown in Table 2. RV dysfunction was associated with an increased risk of in-hospital mortality, regardless of its severity (with RV dysfunction of all grades: odds ratio [OR] 2.15; 95% confidence interval [CI] 1.99–2.32 and with moderate to severe RV dysfunction: OR 1.61; 95% CI 1.49–1.74). The marginal effect of RV dysfunction among patients with LV dysfunction was relatively small compared with that among patients without LV dysfunction.

**Table 2:** Propensity score weighting analyses for predicting in-hospital mortality.

	Adjusted OR (95% CI)	*P*
**Total patients who underwent TTE**		
RV dysfunction	2.15 (1.99–2.32)	< 0.001
Moderate to severe RV dysfunction	1.61 (1.49–1.74)	< 0.001
**Patients with LV systolic dysfunction**		
RV dysfunction	1.76 (1.54–2.01)	< 0.001
Moderate to severe RV dysfunction	1.52 (1.34–1.73)	< 0.001
**Patients without LV systolic dysfunction**		
RV dysfunction	3.31 (2.99–3.66)	< 0.001
Moderate to severe RV dysfunction	3.44 (3.09–3.83)	< 0.001

*CI* confidence interval, *LV* left ventricular, *OR* odds ratio, *RV* right ventricular, *TTE* transthoracic echocardiography.

### Right ventricular dysfunction and outcomes

The results of multivariate regression analysis are shown in Table 3. RV dysfunction was significantly associated with in-hospital mortality (adjusted OR 2.19; 95% CI 1.91–2.53; *P* < 0.001), and the estimated effect size was greater than that for LV dysfunction. The increasing trend of the blood lactate level (i.e., progression of systemic circulatory failure) was not associated with LV dysfunction (adjusted OR 0.80), but was associated with RV dysfunction (adjusted OR 1.31). LV dysfunction and RV dysfunction were associated with lethal arrhythmia, but the adjusted OR of RV dysfunction was higher than that of LV dysfunction (adjusted ORs 2.19 vs. 1.13, respectively). Interaction analysis showed that respiratory failure did not modify the relationship between RV dysfunction and in-hospital mortality (*P* for interaction = 0.538).

**Table 3:** Multivariate logistic regression analyses.

	OR (95% CI)	*P*
**In-hospital mortality**		
RV dysfunction	2.19 (1.91–2.53)	< 0.001
LV systolic dysfunction	0.69 (0.60–0.79)	< 0.001
SOFA score	1.21 (1.19–1.23)	< 0.001
Norepinephrine, mcg/kg/min*	1.27 (1.20–1.36)	< 0.001
**Increased lactate level**		
RV dysfunction	1.31 (1.14–1.50)	< 0.001
LV systolic dysfunction	0.80 (0.70–0.91)	< 0.001
SOFA score	1.12 (1.10–1.14)	< 0.001
Norepinephrine, mcg/kg/min*	1.44 (1.36–1.53)	< 0.001
**Lethal arrhythmias (VT or Vf)**		
RV dysfunction	2.19 (1.34–3.57)	< 0.001
LV systolic dysfunction	1.13 (0.69–1.84)	< 0.001
SOFA score	1.17 (1.10–1.25)	< 0.001
Norepinephrine, mcg/kg/min*	0.90 (0.72–1.12)	< 0.001

*CI* confidence interval, *LV* left ventricular, *OR* odds ratio, *RV* right ventricular, *SOFA* Sequential Organ Failure Assessment, *Vf* ventricular fibrillation, *VT* ventricular tachycardia.

*For every 0.1 mcg/kg/min increase. The doses of other catecholamines were converted into norepinephrine equivalent values and were added.

## Discussion

The two main findings of the present study were that, in patients with septic shock, (1) RV dysfunction predicted in-hospital mortality, lethal arrhythmia, and circulatory insufficiency independently of LV systolic function and (2) visual assessment of RV dysfunction effectively identified the risk of mortality. Our findings may provide rationale for visually assessing RV dysfunction and they support prior studies that showed an association between RV dysfunction and mortality in patients with sepsis or septic shock^11–14^. Moreover, this study may add value to hemodynamic management of septic shock using point-of-care ultrasound.

In this study, RV dysfunction was associated with increased circulatory insufficiency and lethal arrhythmia, with a larger effect than LV systolic dysfunction. Our finding of a relationship between RV dysfunction and mortality is consistent with several previous reports, as well as with a previous study, which showed that LV systolic dysfunction was not associated with the prognosis^13,14,25^. We speculate that RV dysfunction causes secondary cardiogenic shock accompanied by septic shock, which exacerbates systemic perfusion, and ventricular arrhythmias are more likely to occur as complications of cardiogenic shock. A previous study identified the presence of RV dysfunction in patients with cardiac disease as an independent predictor of sudden cardiac death and lethal arrhythmias, irrespective of LV ejection fraction^26^, while another study found that RV ejection fraction was incremental to LV ejection fraction for the prediction of lethal arrhythmic events in patients with LV systolic dysfunction^27^. The results of the current study support these previous findings.

This study also showed that LV dysfunction and RV dysfunction were positively correlated in terms of severity. Additionally, biventricular dysfunction was found in 64% of patients with RV dysfunction, which is similar to findings in a previous study^14^. In contrast, a dilated RV tended to be associated with a normal or relatively small LV size. The relationship between a severely dilated right ventricle and a small or normal LV (i.e., an increased visual RV/LV ratio) suggests that diastolic dysfunction of the left ventricle is exacerbated because of RV dilation, even though LV contractility is preserved^28^. Conversely, acute LV dilation occurs as a compensatory response to depression of LV systolic function to maintain cardiac output before full functional and structural recovery^29,30^. These factors may explain why the estimated effect of RV dysfunction on mortality is greater in patients with isolated RV dysfunction. Consequently, not only LV systolic dysfunction, but also a decrease in RV function and relative expansion of the right ventricle compared with the left ventricle could be important findings of SCM in visual evaluation of echocardiography.

RV dysfunction is caused by systemic inflammation or specific interventions to increase RV afterload and preload (e.g., positive end-expiratory pressure and fluid administration). This reduces the pulmonary circulation and results in reduced cardiac output from the left ventricle^31–33^. In this study, the presence of RV dysfunction was associated with a lower cardiac output status. Furthermore, the pulmonary artery pulsatility index and RV stroke work index were significantly increased in patients with preserved RV function, while they appeared to be absent in patients with RV dysfunction. These results imply that a depressed right ventricle is unable to meet the surge in demand for LV preload, resulting in low cardiac output.

Although excessive fluid loading can clearly induce RV dysfunction, the optimal or excessive fluid infusion is difficult to define because appropriate fluid administration depends on the fluid volume status based on each patient's pretreatment background. Since this study was a retrospective observational study, the timing of TTE and infusion strategy were not standardized. On the other hand, there was no significant difference in the trend of infusion administration between patients with and without RV dysfunction. This suggests that the main trigger of RV dysfunction is not due to differences in infusion strategies.

Visual assessment of RV function by echocardiography is simpler than assessment of other indices, and it is rapid, noninvasive, and suitable for assessing hemodynamics in patients with septic shock^11–14^. We demonstrated that visual assessment of RV dysfunction sufficiently identified an association with increased risk of mortality in patients with septic shock. There is growing evidence of the requirement for mechanical circulatory support in patients with refractory septic shock who have unstable hemodynamics^34–36^. Although visual assessment of RV function by echocardiography is simple, it has low interobserver variability and correlates well with hemodynamic parameters, even compared with assessment by magnetic resonance imaging^15,16^. In the present study, we found that visually assessed RV dysfunction was correlated with trends of hemodynamic parameters as monitored by a pulmonary artery catheter. Notably, despite the fact that the quality of TTE was suboptimal in 38% of patients, the OR of visually assessed RV dysfunction for in-hospital mortality was 2.15. This value is similar to the results of prior studies^13,14^. This result suggests that visual assessment is sufficient for evaluating clinically important RV dysfunction in patients with septic shock. This is the case even when detailed evaluation of right heart function by TTE parameters, such as the fractional area change or tricuspid annulus systolic plane excursion, is difficult to perform. In contrast, however, the marginal odds of moderate to severe RV dysfunction for mortality were similar to those of all grades of RV dysfunction. This finding suggests that visual assessment of RV dysfunction may maintain adequate prognostic capacity without considering its severity.

This study has several limitations. First, this was a retrospective study of a relatively heterogeneous group of patients whose data were extracted from electronic medical records in a large clinical database. Therefore, indication bias may have occurred in patients in whom TTE was performed. In addition, we included patients with a high median SOFA score. Our findings were not extrapolated to the less severe population. Second, although patients with obvious heart disease were excluded, the possibility that they had chronic right heart dysfunction cannot be completely ruled out because there were no records of previous echocardiographic findings. Finally, some variables that were not captured in the database may account for residual confounding. An example of this possible confounding is that pulmonary thromboembolism was not ruled out by contrast-enhanced computed tomography in all cases. Additionally, the possibility that RV dysfunction might have been caused by factors other than sepsis could not be excluded. Further prospective research is required to confirm our results.

## Conclusions

In patients with septic shock, RV dysfunction is associated with increased risks of in-hospital mortality, lethal arrhythmias, and circulatory insufficiency independently of LV dysfunction. Visual assessment of RV dysfunction using echocardiography may thus help to identify the short-term prognosis of patients with septic shock by reflecting hemodynamic status.

## Supplementary Information

Supplementary Information.

## Data Availability

The datasets used and analyzed during the current study are available from the corresponding author on reasonable request.
