# Supplementary material for: Clinical impact of visually assessed right ventricular dysfunction in patients with septic shock
Source: Sci Rep. 2021 Sep 22;11:18823. doi: 10.1038/s41598-021-98397-8 (PMC8458318; doi:10.1038/s41598-021-98397-8)
Supplement: Supplementary file 1 — Supplementary Information. [file 41598_2021_98397_MOESM1_ESM.pdf]

Supplementary Information

Clinical Impact of Visually Assessed Right Ventricular Dysfunction in Patients with Septic Shock

Hiroaki Hiraiwa, Daisuke Kasugai, Masayuki Ozaki, Yukari Goto, Naruhiro Jingushi, Michiko Higashi, Kazuki Nishida, Toru Kondo, Kenji Furusawa, Ryota Morimoto, Takahiro Okumura, Naoyuki Matsuda, Shigeyuki Matsui, Toyoaki Murohara

Supplementary Figure S1. Directed acyclic graph showing the causal relationship between right ventricular dysfunction and mortality.

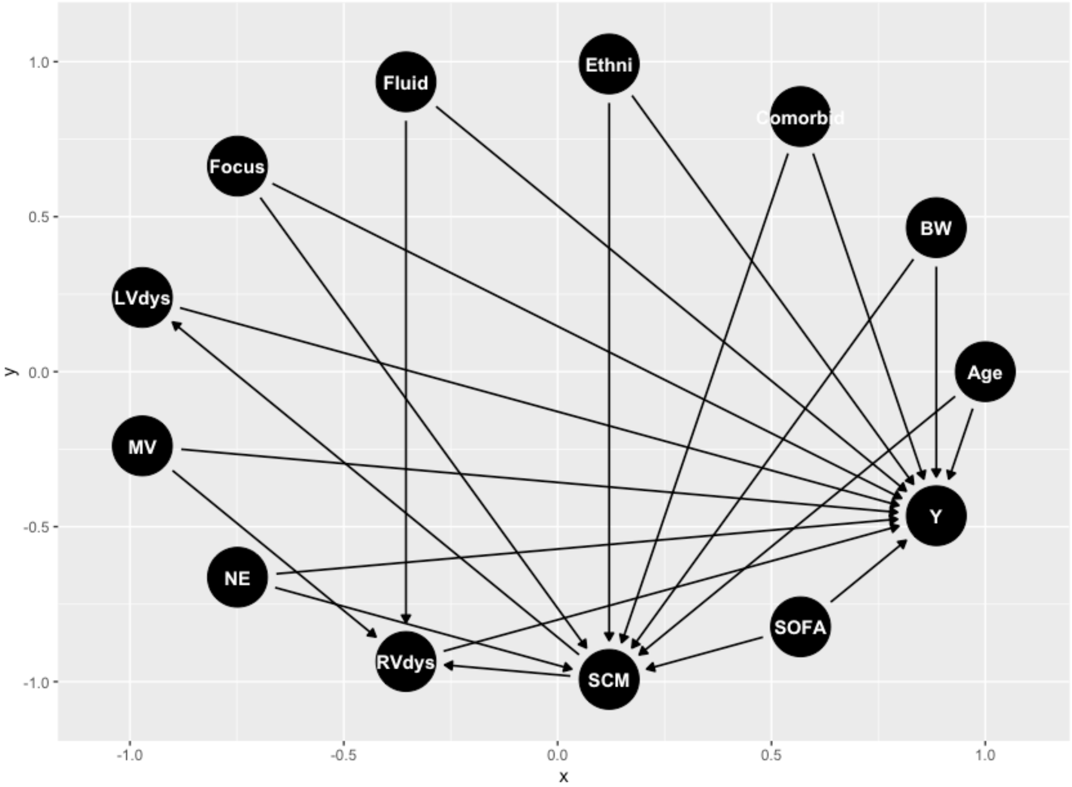

SCM, septic cardiomyopathy; RVdys, right ventricular dysfunction; LVdys, left ventricular dysfunction; MV, mechanical ventilation; BW, body weight; SOFA, Sequential Organ Failure Assessment score; Comorbid, comorbidities; NE, norepinephrine-equivalent dose of catecholamines; Ethni, ethnicity; Focus, focus of infection; Y, outcome.

Supplementary Table S1. Numbers of patients with missing data.

Variable	Number of patients (%)
Age	0 (0)
Ethnicity	0 (0)
Site of infection	0 (0)
Body weight	2 (0.3)
Fluid administration	11 (2)
Creatinine	0 (0)
Platelet	0 (0)
GCS score	3 (0.6)
Bilirubin	56 (10)
Norepinephrine	0 (0)
Lactate	0 (0)
PEEP	87 (16)
P/F ratio	76 (14)
RV function	81 (15)
LV function	15 (3)

GCS, Glasgow Coma Scale; LV, left ventricular; PEEP, positive end-expiratory pressure; RV, right ventricular

Supplementary Table S2. Echocardiographic parameters.

TTE parameters	
Adequate quality, (N=)	
LVEF, % (N = 436)	55 (50-55)
LV systolic function (N = 505)	
Preserved	320 (63.3)
Mildly depressed	47 (9.3)
Moderately depressed	44 (8.7)
Severely depressed	88 (17.4)
Depressed (severity not documented)	6 (1.2)
LV size (N = 450)	
Small	32 (7.1)
Normal	381 (84.7)
Mildly dilated	9 (2.0)
Moderately dilated	18 (4.0)
Severely dilated	4 (0.9)
Dilated (severity not documented)	6 (1.3)
RV function (N = 463)	
Normal	309 (66.7)
Mildly depressed	30 (6.5)
Moderately depressed	22 (4.8)
Severely depressed	37 (8.0)
Depressed (severity not documented)	65 (14.0)
RV size (N = 456)	
Small	5 (1.0)
Normal	297 (65.1)
Mildly dilated	55 (12.1)
Moderately dilated	23 (5.0)
Severely dilated	20 (4.4)
Dilated (severity not documented)	56 (12.3)

Data are presented as median (interquartile range) or n (%).

LV, left ventricular; LVEF, left ventricular ejection fraction; RV, right ventricular; TTE, transthoracic echocardiography
